# The impact of healthcare worker influenza vaccination on nosocomial influenza in a tertiary hospital: an ecological study

**DOI:** 10.1186/s12913-020-05490-1

**Published:** 2020-07-10

**Authors:** Wycliffe Enli Wei, Stephanie Fook-Chong, Wen Kai Chen, Maciej Piotr Chlebicki, Wee Hoe Gan

**Affiliations:** 1 Health Services Research Unit, Singapore General Hospital, Singapore, Singapore; 2 Department of Occupational and Environmental Medicine, Singapore General Hospital, Singapore, Singapore; 3 Duke-NUS Medical School, National University of Singapore, Singapore, Singapore; 4 Department of Infectious Diseases, Singapore General Hospital, Singapore, Singapore; 5 Saw Swee Hock School of Public Health, National University of Singapore, Singapore, Singapore

**Keywords:** Influenza, Vaccination, Health personnel, Healthcare-associated, Cross infection, Vaccination coverage

## Abstract

**Background:**

To protect hospitalized patients, who are more susceptible to complications of influenza, seasonal influenza vaccination of healthcare workers (HCW) has been recommended internationally. However, its effectiveness is still being debated. To assess the effectiveness of HCW influenza vaccination, we performed an ecological study to evaluate the association between healthcare worker influenza vaccination and the incidence of nosocomial influenza in a tertiary hospital within Singapore between 2013 and 2018.

**Methods:**

Nosocomial influenza was defined as influenza among inpatients diagnosed 7 days or more after admission by laboratory testing, while healthcare worker influenza vaccination rate was defined as the proportion of healthcare workers that was vaccinated at the end of each annual seasonal vaccination exercise. A modified Poisson regression was performed to assess the association between the HCW vaccination rates and monthly nosocomial influenza incidence rates.

**Results:**

Nosocomial influenza incidence rates followed the trend of non-nosocomial influenza, showing a predominant mid-year peak. Across 2,480,010 patient-days, there were 256 nosocomial influenza cases (1.03 per 10,000 patient-days). Controlling for background influenza activity and the number of influenza tests performed, no statistically significant association was observed between vaccination coverage and nosocomial influenza incidence rate although a protective effect was suggested (IRR 0.89, 95%CI:0.69–1.15, *p* = 0.37).

**Conclusion:**

No significant association was observed between influenza vaccination rates and nosocomial influenza incidence rates, although a protective effect was suggested. Aligning local HCW vaccine timing and formulation to that of the Southern Hemisphere may improve effectiveness. HCW vaccination remains important but demonstrating its effectiveness in preventing nosocomial influenza is challenging.

## Background

Influenza causes significant morbidity and mortality in older adults and in the presence of comorbidities [1]. To protect hospitalized patients who are more susceptible to complications of influenza, seasonal influenza vaccination of healthcare workers (HCW) has been recommended by many national bodies and has been strongly encouraged if not required in many hospitals internationally [1].

There has been some evidence of its effectiveness in reducing nosocomial influenza, largely in older patients in long-term care facilities [2]. However, its effectiveness is still being debated with a recent review showing no effect on the incidence of lab-confirmed influenza [3].

At an institution-level, there have been few studies that evaluated the effectiveness of institution-wide influenza vaccination on nosocomial influenza incidence rates, most of which are from the northern hemisphere [4, 5]. These commonly report and analyze aggregated annual influenza proportions. Studies in tropical countries, where influenza activity is year-round and show multiple peaks [6], and those that report incidence rates are lacking.

### Aim

As a large tertiary hospital located in the tropics, we aimed to evaluate the association between HCW influenza vaccination and nosocomial influenza incidence rates. We hypothesized that increasing seasonal influenza vaccination coverage amongst clinical HCWs is associated with reduced nosocomial influenza incidence rates.

## Methods

### Study design

This ecological study evaluates the association between HCW seasonal influenza vaccination rates and corresponding nosocomial influenza rates in a large tertiary hospital in Singapore from October 2013 to October 2018. The tertiary hospital is one of Singapore’s largest with more than 1700 beds, comprising 46 adult clinical specialties and having a staff strength of almost 9900. It admits around 80,000 patients annually, accounting for almost one-quarter of all inpatient admissions nationally.

### Data sources

De-identified data on (1) all influenza-related laboratory test results (including PCR, antigen and viral culture tests), (2) dates of admission of patients with influenza-related tests and (3) monthly aggregate of inpatient days were extracted from the hospital’s enterprise data repository. This repository consolidates healthcare data from multiple sources including hospital electronic health records and administrative systems [1, 2].

Staff influenza vaccination numbers were based on the records of the hospital staff vaccination programme.

National influenza surveillance data was obtained from the World Health Organisation FluNet, Global Influenza Surveillance and Response System [3]. This represents Singapore’s national data derived from sentinel surveillance. The proportion of specimens collected that tested positive for influenza was used as a measure of background influenza activity.

### Healthcare worker influenza vaccination

Annual seasonal influenza vaccines were offered to hospital staff free of charge. Uptake was voluntary but strongly encouraged by the hospital management. The trivalent influenza vaccine was administered till 2017, after which it was replaced by the quadrivalent vaccine. The vaccines follow the northern hemisphere schedule except in 2018, during which the southern hemisphere formulation was administered between the months of May and June. The choice of vaccine formulation, and thus timings, may be advised by the Singapore Ministry of Health based on its coverage of influenza strains. Where there was no preference for either vaccine formulation in a year, vaccination exercise timing was decided based on organizational factors. Vaccination exercises span 2 to 3 months (months of October to December in 2013 to 2016, July to September in 2017, and May to June in 2018).

The healthcare worker influenza vaccination rate was defined as the proportion of healthcare workers that was vaccinated at the end of each annual seasonal vaccination exercise. The vaccination rate was applied from the starting month of the vaccination exercise for that year, till the month before the start of the subsequent vaccination exercise. Vaccination coverage of clinical HCWs (doctors, nurses and allied health) was used in the main analysis.

### Nosocomial influenza incidence rate

The case definition of nosocomial influenza is a patient with a positive laboratory test for influenza performed 7 days or more after admission, while remaining cases of influenza were deemed non-nosocomial. Laboratory testing for influenza in inpatients was based on clinical need in the diagnostic work-up of pneumonia and respiratory disease. Cut-off times based on clinical symptoms have been proposed to be between 48 and 72 h [7], while a cut-off of 7 days has been used for laboratory-based diagnosis [8].

### Potential confounders

Institutional non-nosocomial influenza incidence rates were used to control for confounding due to changes in background influenza patterns. This was calculated based on laboratory-positive cases that did not meet the nosocomial case definition. National influenza surveillance data were also obtained for this purpose. The number of inpatients who were tested for influenza was also collected as a possible confounder.

### Statistical analysis

Modified Poisson regression was performed to assess the effect of HCW vaccination rates on monthly nosocomial influenza incidence rates (with number of inpatient-days as offset variable) while controlling for the above-mentioned confounders. Sensitivity analyses were also performed. We repeated the analyses using vaccination coverage of all hospital staff and by varying the time criteria for the definition of nosocomial influenza. Also, we examined if using national influenza surveillance data to control for background influenza activity in addition to hospital non-nosocomial influenza incidence rates changed our results. Appropriateness of the model was assessed by plots of the autocorrelation and partial autocorrelation functions, as well as a residual plot. STATA 14.0 (Stata Corp, College Station, Tx, USA) was used for statistical analysis.

## Results

### Nosocomial influenza cases and incidence

Between October 2013 to October 2018, there were 256 cases of nosocomial influenza over 2,480,010 patient-days, corresponding to an incidence rate of 1.03 cases per 10,000 patient-days. Among all diagnoses of influenza, 7.1% fulfilled the criteria of nosocomial influenza (Table 1).

**Table 1:** Aggregate statistics of nosocomial influenza* and healthcare worker vaccination rates from Oct 2013 to Oct 2018

Period	Oct 2013 - Sep 2014	Oct 2014 - Sep 2015	Oct 2015 - Sep 2016	Oct 2016 - Jun 2017	Jul 2017 - Apr 2018	May 2018 - Oct 2018	Total
**Patient Days**	477,833	494,391	486,375	364,511	408,613	248,287	2,480,010
**Number of Inpatients Tested for Influenza**^**a**^	4242	4741	6249	4054	4577	2417	26,280
**Number of Influenza Cases**^**b**^	623	694	856	621	568	241	3603
**Nosocomial Influenza Cases**^**b**^	36	71	48	52	31	18	256
**Incidence Rate (per 10,000 patient-days)**	0.75	1.44	0.99	1.43	0.76	0.72	1.03
**Proportion of Nosocomial Cases**	5.8%	10.2%	5.6%	8.4%	5.5%	7.5%	7.1%
**Overall Vaccination Rate**	52.1%	48.8%	50.9%	51.3%	70.9%	77.1%	–
**Clinical Staff Vaccination Rate (Doctors, Nurses, Allied Health Professionals)**	56.3%	49.4%	54.7%	52.9%	72.6%	78.9%	–
**Non-Clinical Staff Vaccination Rate (Ancillary and Administrative Staff)**	40.9%	47.1%	42.5%	48.1%	67.2%	72.6%	–

*Nosocomial influenza is defined as influenza diagnosed by laboratory tests 7 days or more after admission

^a^Enumerated by episodes of inpatient admission

^b^Laboratory-confirmed

**Fig. 1:**
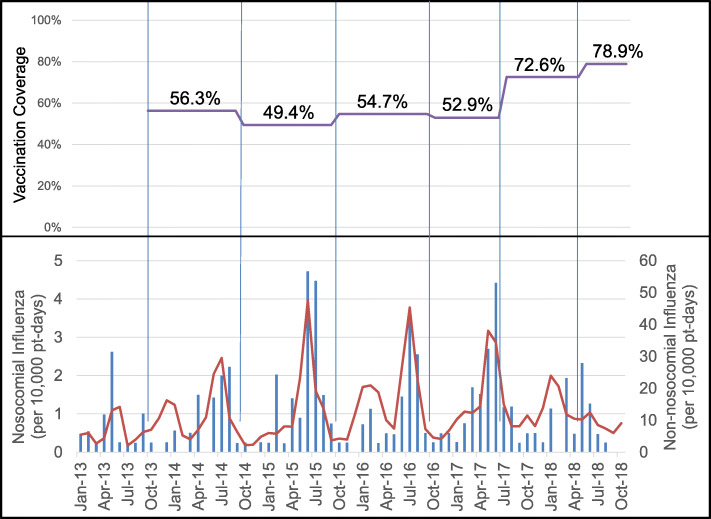
Incidence rates of lab-diagnosed influenza* (bottom panel) with clinical healthcare worker vaccination coverage (top panel), January 2013 to October 2018. In the bottom panel, the bars represent nosocomial influenza incidence rates corresponding to the left y-axis, while the line represents non-nosocomial influenza incidence rates corresponding to the right y-axis. * Influenza cases diagnosed 7 days or more after admission are considered to be nosocomial infections, while those diagnosed 7 days or less are considered to be non-nosocomial or community-acquire

The incidence pattern of non-nosocomial influenza shows a predominant mid-year peak. Smaller peaks corresponding to the northern hemisphere influenza season are also seen. In 2018, the mid-year non-nosocomial influenza activity was observed to be lower compared to other years. Generally, nosocomial influenza incidence rates followed the trend of non-nosocomial influenza (Fig. 1).

### Vaccination trends

Between October 2013 to June 2017, overall influenza vaccination coverage hovered between 48.8 to 52.1%. The vaccination exercise in July 2017 saw good uptake of 70.9% and an even greater coverage of 77.1% was attained in mid-2018.

Among healthcare staff, nurses consistently had the highest vaccination coverage, followed by ancillary staff. In contrast, doctors had lower vaccination coverage compared to other vocations.

### Association between vaccination rates and nosocomial influenza

In our final model that adjusted for the institutional parameters of non-nosocomial (background) influenza incidence and the volume of influenza tests performed, we did not observe a significant association between the proportion of HCW vaccinated and the nosocomial influenza incidence, although the results suggested a protective effect. For a 10% increase in vaccination rate of clinical HCWs, there was an estimated 11% decrease (Incidence Rate Ratio (IRR) 0.89, 95%CI:0.69–1.15, *p* = 0.37) in the incidence rate of nosocomial influenza (Table 2).

**Table 2:** Association between lab-defined nosocomial influenza and influenza vaccination rate

Variables	Crude IRR	95% CI	***p***-value	Adjusted IRR	95% CI	***p***-value
Clinical HCW Vaccination Rate (per 10% increase)	0.79	(0.58, 1.07)	0.128	0.89	(0.69, 1.15)	0.37
Number of Admissions in which Influenza Testing was Performed	1.01	(1.00, 1.01)	< 0.001	1.00	(1.00, 1.01)	0.24
Non-Nosocomial Influenza Incidence Rate (per 1 in 10,000 patient-days)	1.05	(1.04, 1.07)	< 0.001	1.04	(1.00, 1.07)	0.027
Time (months)	1.00	(0.99, 1.02)	0.50	–	–	–

A sensitivity analysis with vaccination rates defined as low or high using a cut-off of 70%, as well as a sensitivity analysis using overall hospital staff’s vaccination rates show similar associations between vaccination coverage and nosocomial influenza incidence (Table 3). We noted that overall hospital staff vaccination rates had a weaker association with nosocomial influenza than when clinical HCW rates were used. When the threshold for the diagnosis of nosocomial influenza was reduced to 4 days, the association was also weakened to an IRR of 0.94 (95%CI: 0.79–1.12). We also assessed further controlling for background trends by using national sentinel influenza surveillance data in addition to the hospital non-nosocomial influenza incidence rate; this did not alter results and provided inferior models. Residual diagnostics of the main model showed no significant autocorrelation of data in the series and thus, the regression model is a good fit for our data.

**Table 3:** Estimated effect of vaccination on nosocomial influenza under variations of the analysis (sensitivity analyses)

Sensitivity Analyses	Effect of Vaccination on Nosocomial Influenza Rate, Adjusted IRR^a^
***By different groups of healthcare workers (IRR per 10% increase in vaccine coverage)***	
Overall	0.93 (0.73–1.18)
Clinical Healthcare Workers	0.89 (0.69–1.15)
Nursing	0.88 (0.67–1.16)
***By categorization of vaccination rate***	
Clinical Healthcare Workers (IRR per 10% increase in vaccine coverage)	0.89 (0.69–1.15)
Clinical Healthcare Workers (IRR of coverage ≥70% vs <70%)	0.83 (0.50–1.40)
***By time criteria of nosocomial influenza diagnosis (IRR per 10% increase in vaccine coverage)***	
Cut-off of 7 days or more	0.89 (0.69–1.15)
Cut-off of 4 days or more	0.94 (0.79–1.12)
Cut-off of 3 days or more	0.96 (0.82–1.13)
***By inclusion of national influenza sentinel surveillance information***^**b**^***(IRR per 10% increase in vaccine coverage)***	
Without national surveillance information	0.89 (0.69–1.15)
With national surveillance information	0.89 (0.69–1.15)

^a^Adjusted for number of admissions in which influenza testing was performed and non-nosocomial influenza incidence rate

^b^Proportion of sentinel surveillance samples testing positive for influenza

## Discussion

Our study did not show a statistically significant association between vaccination coverage and nosocomial influenza incidence rate, although the results suggest a protective effect – a 10% increase in vaccination coverage was estimated to correspond to 11% decrease in nosocomial influenza incidence rate (*p* = 0.37). It was also observed that influenza activity in Singapore predominantly followed the Southern Hemisphere influenza seasonal pattern between 2013 and 2018.

Similar studies, interestingly few, show that HCW vaccination coverage is associated with reduced nosocomial influenza incidence [4, 5]. Our study observed a similar trend, but statistical significance was not reached despite a relatively large number of nosocomial influenza cases. The effect size observed in this study, when measured by proportion of nosocomial influenza (not shown) is similar to that of a study performed in cancer patients [4].

A mismatch in the Northern Hemisphere timing of HCW vaccination and predominantly Southern Hemisphere seasonal peaks in influenza activity in our study may have reduced the effect size observed and contributed to the lack of statistical significance. Furthermore, virulence, vaccine efficacy and the match of vaccine strains vary between influenza seasons and may contribute to significant variation.

Notwithstanding this, the results are encouraging. Firstly, the observed effect size is clinically significant. Secondly, dose-effect relationships were observed in the sensitivity analyses. When non-clinical staff were included in the calculation of vaccine coverage, the association observed was weakened. Decreasing the diagnostic specificity of nosocomial influenza by using less stringent classification thresholds (days from admission to laboratory diagnosis) resulted in a weaker association. This is consistent with a protective effect expected of HCW influenza vaccination on nosocomial influenza.

Singapore is located 1.3°N and was deemed to have year-round influenza activity and varying peak periods [6, 9]. WHO had also recommended Singapore to adopt the Northern Hemisphere influenza vaccine formulation [10]. In contrast, our hospital-based data from 2013 to 2018 indicated that influenza activity, both nosocomial and community-acquired, predominantly shows southern hemisphere seasonal peaks. A local study concurs, reporting that “severe epidemics were more commonly observed around middle of the year [9]”.

In view of the findings, a Southern Hemisphere vaccination timing should be considered in our hospital, if not nationally. Nevertheless, the seasonal profile of influenza activity may not be stable in the tropics and continued influenza surveillance is needed. Given that tropical countries face year-round influenza activity, selection of vaccine formulation should also be guided by strain coverage. This requires evaluation if differences in vaccine strains are significant enough to prefer one over another. To achieve good coverage of circulating strains, there may even be years where at-risk populations are recommended to be vaccinated with both the Southern and Northern Hemisphere vaccines in the same year, as was the case in 2019 [11]. Such considerations may influence vaccination timing in some years. However, where there is no such preference between vaccine formulations in a year, our data suggests that a Southern Hemisphere vaccination timing may better match with influenza activity peaks.

The ecological study design has limitations. Potential unmeasured confounders such as improved infection control measures coinciding with improved vaccination uptake may give rise to false associations. Nevertheless, an ecological study is practical and feasible compared to other study designs in assessing the effectiveness of an institutional influenza vaccination program. Particularly, it is challenging to determine an individual patient’s exposure to unvaccinated HCWs.

The study likely underestimates the true incidence of nosocomial incidence. The definition of nosocomial influenza of laboratory diagnosis at 7 days or more from admission yields specificity but may underestimate the incidence given that the incubation period is between 1 to 4 days. Underestimation may also arise from passive surveillance of laboratory data rather than active sampling of inpatients for influenza. As there was no post-discharge surveillance, infected cases who became symptomatic after discharge may not be detected and contributes to underestimation.

Nevertheless, our study is well-sized with 256 cases over 2,480,010 patient-days. We also analyze monthly nosocomial influenza incidence rates, while other studies often use the annual proportions of nosocomial influenza amongst all influenza diagnosed which may be difficult to interpret and compare [4, 5]. This study also presents evidence and a perspective from a tropical country with differing influenza seasonal patterns and less certain vaccination timings to match peak influenza activity.

## Conclusion

We observed a no statistically significant association between influenza vaccination rates and nosocomial influenza incidence rates, although a protective effect was suggested. Based on observed trends, aligning the local vaccine timing to that of the Southern Hemisphere may improve effectiveness.

## Data Availability

The data that support the findings of this study are available from the Singapore General Hospital, but restrictions apply to the availability of these data, which were used under license for the current study, and so are not publicly available. Data are however available from the authors upon reasonable request and with permission of the Singapore General Hospital.

## Abbreviations

HCW: Healthcare Worker
IRR: Incidence Rate Ratio
